# Childhood body size and pubertal timing in relation to adult mammographic density phenotype

**DOI:** 10.1186/s13058-017-0804-y

**Published:** 2017-02-07

**Authors:** Minouk J. Schoemaker, Michael E. Jones, Steven Allen, Jean Hoare, Alan Ashworth, Mitch Dowsett, Anthony J. Swerdlow

**Affiliations:** 1 Division of Genetics and Epidemiology, The Institute of Cancer Research, 15 Cotswold Road, London, SM2 5NG UK; 2 Department of Diagnostic Radiology, Royal Marsden Hospital NHS Foundation Trust, Downs Road, Sutton, SM2 5PT UK; 3 UCSF Helen Diller Family Comprehensive Cancer Center, San Francisco, CA 94158 USA; 4 Division of Breast Cancer Research, The Institute of Cancer Research, 237 Fulham Road, London, SW3 6JB UK; 5 Breast Cancer Now Research Centre, The Institute of Cancer Research, 237 Fulham Road, London, SW3 6JB UK; 6 Academic Department of Biochemistry, Royal Marsden NHS Foundation Trust, 203 Fulham Road, London, SW3 6JJ UK; 7 Division of Molecular Pathology, The Institute of Cancer Research, 237 Fulham Road, London, SW3 6JB UK

**Keywords:** Body weight, Body height, Breast neoplasms, Cross-sectional study, Mammographic density, Puberty, Adolescent

## Abstract

**Background:**

An earlier age at onset of breast development and longer time between pubertal stages has been implicated in breast cancer risk. It is not clear whether associations of breast cancer risk with puberty or predictors of onset of puberty, such as weight and height, are mediated via mammographic density, an important risk factor for breast cancer.

**Methods:**

We investigated whether childhood body size and pubertal timing and tempo, collected by questionnaire, are associated with percentage and absolute area mammographic density at ages 47–73 years in 1105 women recruited to a prospective study.

**Results:**

After controlling for adult adiposity, weight at ages 7 and 11 years was strongly significantly inversely associated with percentage and absolute dense area (*p* trend <0.001), and positively associated with absolute non-dense area. Greater height at age 7, but not age 11, was associated with lower percentage density (*p* trend = 0.016). Later age at menarche and age at when regular periods were established was associated with increased density, but additional adjustment for childhood weight attenuated the association. A longer interval between thelarche and menarche, and between thelarche and regular periods, was associated with increased dense area, even after adjusting for childhood weight (*p* trend = 0.013 and 0.028, respectively), and was independent of age at pubertal onset.

**Conclusions:**

Greater prepubertal weight and earlier pubertal onset are associated with lower adult breast density, but age at pubertal onset does not appear to have an independent effect on adult density after controlling for childhood adiposity. A possible effect of pubertal tempo on density needs further investigation.

**Electronic supplementary material:**

The online version of this article (doi:10.1186/s13058-017-0804-y) contains supplementary material, which is available to authorized users.

## Background

Breast cancer is the most common type of cancer in women, and the incidence has been increasing [1]. The distribution of risk factors for breast cancer have changed over time, such as increasing obesity [2] and height [3] and declining age at onset of puberty [4]. Mammographic density is one of the strongest risk factors for breast cancer [5], with fourfold to fivefold increases in risk in those with at least 75% density. Density reflects variations in the tissue composition of the breast, with the dense area representing collagen and epithelial cells and the non-dense area representing adipose tissue. The amount of dense tissue is thought to be the aetiologically relevant parameter related to breast cancer risk, although percentage density (amount of dense area over total breast area, expressed as a percentage) has been found to be a stronger risk predictor than absolute dense area, and whether there is an independent protective role of non-dense tissue is still unclear [6].

While earlier menarche is an established risk factor for breast cancer, we recently reported that other pubertal stages also contribute to the risk, ﻿based on data from a large prospective cohort study. Earlier breast development (thelarche), and a longer interval between thelarche and menarche were independently associated with a 20–30% increase in breast cancer risk. Risk was also increased in women in whom menses became regular and adult height was reached at an earlier age [7].

Whether pubertal associations with breast cancer risk are mediated through mammographic density is unclear. Breast tissue composition has been hypothesised to be determined by genetic factors and growth and development in early life [8]. During pubertal development breast tissue undergoes substantial cellular proliferation and is subject to hormonal surges and it is possible that the age at which such growth occurs and the speed of the growth affects breast density and cancer risk. Previous studies of the association between puberty and adult breast density have mostly investigated menarche [9–16], and one study previously reported on linear growth and Wolfe’s grading of density [14]. To our knowledge, no previous studies have addressed the association between pubertal stages other than menarche, or time intervals between pubertal stages, and quantitative measures of adult density.

Childhood height and adiposity are established predictors of pubertal onset ([17], and childhood height has been associated with greater density in some studies [13, 18]. On the contrary, childhood adiposity has been reported to be inversely associated with mammographic density, although not consistently so, with a recent review concluding that additional research is needed to clarify this complex association [19]. Besides investigating the associations between breast density and puberty or adiposity in their own right, it is of interest to investigate these together so as to evaluate whether the potential association between density and pubertal stage is independent of the effect of adiposity.

We analysed the association between adult mammographic density phenotype and childhood weight and height, and pubertal stages and timing, in a sample of women who participated in a large UK-based prospective cohort study focussed on breast cancer aetiology.

## Methods

### Participants

Study subjects were identified from the Generations Study, a UK-based cohort study with over 113,000 participants, which was designed to investigate breast cancer aetiology [20]. Volunteers completed a postal questionnaire about established and putative breast cancer risk factors and, if willing, donated a blood sample. Participants are contacted approximately every 3 years to collect follow-up information on breast cancer diagnoses and updated risk factor information.

The study subjects in the current analysis are the control subjects included in a nested case-control study of breast cancer occurring within the cohort. One or more controls per case were randomly selected from subjects who had been free of breast cancer for at least as long as the matched case, within strata of the categories of year and age at study entry, ethnicity and the number of days between blood draw and receipt of the blood sample in the laboratory. For women who reported in their questionnaire that they had had a mammogram, the mammograms were requested from the breast cancer screening centre in the UK that matched the self-reported screening location. Under the National Breast Cancer Screening Programme, women have been invited to these centres for routine 3-yearly screening at ages 50–70 years, and this has recently been extended to ages 47–73 years.

The mammographic radiographs from the screening visits were digitised with a VIDAR Diagnostic Pro Plus scanner, which covers an optical density range of 0.0–3.85. With the roll-out of digital mammography in the UK, we increasingly also received digital images in electronic format, but these were excluded from this analysis due to small numbers. The mammograms from the screening visit closest (before or after) to the date of entry to the cohort study at screening ages 47–73 years were selected for this analysis. Percentage mammographic density and absolute dense and non-dense area (in centimetres squared) was determined using Cumulus software [21]. Images were assessed by one observer, blinded to case-control status, who was trained by an experienced breast radiologist (SA). Two mediolateral oblique (MLO) projections per subject were selected for reading. The images were randomly allocated to batches that included repeats, based on which the intra-class correlation coefficient for percent density was 0.93. Analyses were based on the average of the density readings of the projections of the left and right breast.

The baseline questionnaire included information on weight and height relative to peers at age 7 and 11 years, in five categories (e.g. for weight: much thinner, a little thinner, about the same, a little heavier, much heavier or do not remember). It also included information on age at first breast development, at menarche, at establishment of regular cycles and at reaching adult height, based on which the time intervals between stages were computed, and on other breast cancer risk factors including adult height and weight, which were used to compute the participant’s body mass index (BMI). Information on follow-up questionnaires was used to update exposures, where applicable, for women in whom mammography was conducted after they had completed the baseline questionnaire.

### Statistical analysis

We analysed mammographic density parameters in relation to pubertal factors and childhood body size with a linear regression model using density parameters which were square-root-transformed to ensure the normality of the residuals. We derived absolute differences in density parameters between categories of explanatory factors so that effect estimates could be presented as percentage point differences for percent density and in centimetres squared for dense and non-dense area. This was done by back-transforming the coefficients relative to a predetermined reference level of 25% density, 30 cm^2^ dense area and 110 cm^2^ non-dense area, respectively, so that the effect estimates could be directly compared between variables, because the absolute difference would otherwise depend on the average of the density parameter in the reference group. The statistical package Stata 14.0 was used throughout [22]. All reported *p* values are two-sided.

Analyses were adjusted for age at mammography and other mammographic density risk factors possibly associated with childhood body size or pubertal onset: age at first birth and parity, duration of oral contraceptive use, alcohol consumption and physical activity level, menopausal status and, in postmenopausal women, time since menopause and postmenopausal use of oestrogen and progestogen hormone therapy.

In the literature, analyses of percentage density with respect to breast cancer risk are conventionally adjusted for BMI, as the same percentage density for a woman with high BMI does not represent the same amount of dense tissue (thought to be the aetiological parameter with respect to breast cancer risk) than in a woman with low BMI. However, for our analyses of determinants of density, given the correlation between BMI and childhood weight, adjustment for BMI could result in over-adjustment of the association between childhood body size, puberty and density. We therefore conducted the analyses with and without adjusting for BMI, as recommended elsewhere [19]. We also repeated the puberty analyses with additional adjustment for childhood body size to investigate whether the association with puberty is independent of childhood body size. Alcohol consumption, BMI, and physical activity level were assessed in the baseline questionnaire and all other factors were evaluated as closely as possible to the time of the mammogram, using data on calendar years and ages provided in the baseline and follow-up questionnaires.

## Results

Mammograms were retrieved for 81.6% of subjects who were within screening ages 47–73 years at the time of the baseline questionnaire, with the main reasons for non-retrieval being that films were no longer held at the screening centre or lack of detail on the questionnaire to locate the screening centre. A total of 1105 subjects were included in the analysis: their mean age at mammography was 58.9 years, and 80.1% were postmenopausal at the time of the mammogram (Table 1). The median interval between the baseline questionnaire and mammography was 1.0 year. Arithmetic mean values were 22.9% for mammographic density, 28.7 cm^2^ for absolute dense area and 112.9 cm^2^ for non-dense area. Numbers of subjects per category of body size and pubertal factor are provided in Additional file 1: Table S1.

**Table 1:** Characteristics of the study population (subjects with mammographic density data participating in the Generations Study)

	Participants		
Characteristic	Adjusted mean percentage density^a^	Number	Percentage of subjects
*Age at mammogram, years*			
47–54	25.5	303	27.4
55–59	21.3	344	31.1
60–64	18.0	279	25.2
65–73	16.6	179	16.2
*Interval between mammogram and baseline questionnaire*			
≥3 years prior	21.6	47	4.3
2–2.9 years prior	24.3	61	5.5
1–1.9 years prior	24.0	182	16.5
Within 1 year	21.1	554	50.1
1–1.9 years later	19.3	127	11.5
2–2.9 years later	22.0	59	5.3
≥3 years later	17.2	75	6.8
*BMI at baseline questionnaire, kg/m* ^*2*^			
<20	33.0	39	3.5
20–24	25.6	525	47.5
25–29	15.7	378	34.2
≥30	12.1	163	14.8
*Menopausal status at mammogram*			
Postmenopausal	20.5	885	80.1
Premenopausal	24.5	126	11.4
Status not known	23.2	94	8.5
*Parity*			
Nulliparous	24.5	113	10.2
Parous	20.9	992	89.8
*Age at first birth, years*			
<25	19.9	428	38.7
25–29	21.4	414	37.5
≥30	22.4	150	13.6
*Number of births*			
1	19.7	100	9.0
2	21.1	583	52.8
≥3	21.0	309	28.0
*Postmenopausal hormone replacement at time of mammogram*			
Never	21.0	839	75.9
Former	21.0	199	18.0
Current	25.7	67	6.1
Total study population	21.3	1105	100.0

^a^Mean percentage mammographic density (back-transformed to ordinary scale) for average body mass index (BMI) 25 kg/m^2^ and age 58 years at mammogram for all variables, except category of age at mammogram (at BMI 25 kg/m^2^ only) and category of BMI (at age 58 years only)

Women who had been heavier than their peers at age 11 years reported an earlier onset of pubertal stages, consistent with an earlier report from the entire cohort of the Generations Study [23]. Heavier girls also reported longer intervals between thelarche or menarche and attained adult height, higher BMI at study entry, higher non-dense mammographic area and lower percentage and absolute mammographic dense area than those who were lighter (Additional file 1: Table S2). Taller girls had an earlier onset of pubertal stages but there was no difference in the intervals between stages compared with girls who were of similar or of shorter height. Those who were tall at age 11 years were taller in adulthood and had larger non-dense and total mammographic breast area (Additional file 1: Table S3). There was modestly strong correlation between age at thelarche and age at menarche (*r* = 0.74), but weak correlation between other stages (Additional file 1: Table S4).

Weight at ages 7 and 11 years was significantly inversely associated with percentage density and absolute dense area and significantly positively associated with non-dense area (Table 2). These associations were attenuated, but remained statistically significant, after adjusting for adult BMI. A relative increase in weight compared with peers between age 7 and 11 was similarly associated with density parameters but estimates were no longer statistically significant after taking adult BMI into account.

**Table 2:** Difference in adult mammographic density across categories of weight compared with peers at ages 7 and 11 years

		Mammographic density parameters		
			Absolute area	
		Percent density difference,	Dense area	Non-dense
Weight and age	Category	percentage points (95% CI)^a^	difference, cm^2^ (95% CI)^a^	difference, cm^2^ (95% CI)^a^
*Weight relative to peers, age 7 years*				
A:	Thinner	5.6 (3.0, 8.4)	6.1 (3.0, 9.3)	-10.8 (-17.7, -3.6)
	About the same	0.0 (baseline)	0.0 (baseline)	0.0 (baseline)
	Heavier	-4.1 (-7.0, -0.9)	-2.1 (-5.7, 1.8)	17.7 (6.9, 28.9)
	*P* trend^b^	**<0.001**	**<0.001**	**<0.001**
B: +BMI-adjusted	Thinner	4.2 (1.8, 6.7)	5.7 (2.7, 8.9)	-4.6 (-10.3, 1.3)
	About the same	0.0 (baseline)	0.0 (baseline)	0.0 (baseline)
	Heavier	-1.6 (-4.5, 1.5)	-1.1 (-4.8, 2.8)	3.5 (-4.7, 12.0)
	*P* trend^b^	**<0.001**	**<0.001**	**0.050**
*Weight relative to peers, age 11 years*				
A:	Thinner	5.0 (2.4, 7.8)	4.3 (1.3, 7.5)	-12.8 (-19.7, -5.7)
	About the same	0.0 (baseline)	0.0 (baseline)	0.0 (baseline)
	Heavier	-7.0 (-9.3, -4.5)	-6.4 (-9.3, -3.4)	23.3 (14.0, 32.9)
	*P* trend^b^	**<0.001**	**<0.001**	**<0.001**
B: +BMI-adjusted	Thinner	3.0 (0.7, 5.5)	3.8 (0.8, 7.0)	-4.1 (-9.9, 1.9)
	About the same	0.0 (baseline)	0.0 (baseline)	0.0 (baseline)
	Heavier	-4.1 (-6.5, -1.6)	-5.4 (-8.3, -2.2)	5.8 (-1.3, 13.1)
	*P* trend^b^	**<0.001**	**<0.001**	**0.017**
*Change in relative weight age 7 to 11 years* ^c^				
A:	Decrease	0.2 (-4.6, 5.6)	-0.7 (-6.3, 5.5)	2.8 (-12.3, 19.0)
	About the same	0.0 (baseline)	0.0 (baseline)	0.0 (baseline)
	Increase	-6.3 (-9.0, -3.3)	-6.1 (-9.4, -2.6)	24.1 (13.3, 35.3)
	*P* trend^b^	**<0.001**	**0.007**	**<0.001**
B: +BMI-adjusted	Decrease	0.2 (-4.3, 5.1)	-0.3 (-5.9, 5.8)	3.2 (-8.8, 15.9)
	About the same	0.0 (baseline)	0.0 (baseline)	0.0 (baseline)
	Increase	-2.8 (-5.6, 0.2)	-4.4 (-7.9, -0.7)	5.9 (-2.2, 14.2)
	*P* trend^b^	0.11	0.054	0.36

^a^Differences derived with respect to reference levels: 25% for percentage density, 30 cm^2^ for dense area and 110 cm^2^ for non-dense area. Models defined as A: analyses adjusted for age at mammogram (47–50, 50–54, 55–59 (baseline), 60–64, 65–69, 70–73 years), duration of oral contraception use (never (baseline), <5, 10–14, ≥15 years, not known), postmenopausal hormone treatment (never (baseline), former, current/<5, current/5–9, current/≥10 years duration), menopausal status and time since menopause (<5 (baseline), 10–14, 15–19, ≥20, unknown years postmenopausal, not postmenopausal), age at first birth and parity (nulliparous, 10–24 years/1–2, 10–24 years/≥3, 25–29 years/1–2 (baseline), 25–29 years/≥3, 30 years/≥1), alcohol units (none (baseline), 1–4 to ≥25, in 5-unit increments), physical activity (<31 (baseline), 32–55, 56–88, ≥88 metabolic energy equivalents/h/week); B: adjusted for covariates in model A plus body mass index (BMI) (<20.0 (baseline) to >35.0, in 2.5 kg/m^2^ increments). ^b^P trend for linear regression fitted through categories of exposure. ^c^Increase or decrease in category of weight compared with peers between ages 7 and 11 years

The *p*-values in bold are those with *p*<0.05

There was a tendency for taller girls to have lower percentage density and increased non-dense area compared to those who were shorter, even after adjusting for adult adiposity (Table 3), although the association with percentage density was only significant for height at age 7 but not at age 11 years. There was no association with absolute dense area (Table 3) or with change in relative height between age 7 and 11 (Additional file 1: Table S5).

**Table 3:** Difference in adult mammographic density parameters across categories of height compared with peers at ages 7 and 11 years

		Mammographic density parameters		
			Absolute area	
		Percent density difference,	Dense area	Non-dense area
Height and age	Category	percentage points (95% CI)^a^	difference, cm^2^ (95% CI)^a^	difference, cm^2^ (95% CI)^a^
*Height relative to peers, age 7 years*				
A:	Shorter	3.7 (0.9, 6.7)	3.7 (0.4, 7.2)	-10.7 (-18.5, -2.7)
	About the same	0.0 (baseline)	0.0 (baseline)	0.0 (baseline)
	Taller	0.0 (-2.5, 2.6)	1.2 (-1.9, 4.4)	2.5 (-5.4, 10.8)
	*P* trend^b^	**0.027**	0.23	**0.006**
B: +BMI-adjusted	Shorter	2.8 (0.2, 5.5)	3.1 (-0.1, 6.5)	-7.7 (-13.9, -1.3)
	About the same	0.0 (baseline)	0.0 (baseline)	0.0 (baseline)
	Taller	-0.8 (-3.1, 1.6)	0.5 (-2.5, 3.6)	5.6 (-0.7, 12.1)
	*P* trend^b^	**0.016**	0.19	**<0.001**
C: +weight age 11 years	Shorter	1.9 (-0.7, 4.6)	1.8 (-1.4, 5.2)	-6.9 (-13.2, -0.3)
	About the same	0.0 (baseline)	0.0 (baseline)	0.0 (baseline)
	Taller	-0.3 (-2.6, 2.1)	1.2 (-1.8, 4.3)	4.9 (-1.5, 11.4)
	*P* trend^b^	0.16	0.79	**0.002**
*Height relative to peers, age 11 years*				
A:	Shorter	3.0 (0.2, 5.9)	3.3 (0.1, 6.7)	-7.8 (-15.5, 0.3)
	About the same	0.0 (baseline)	0.0 (baseline)	0.0 (baseline)
	Taller	0.6 (-1.9, 3.2)	1.6 (-1.4, 4.7)	1.7 (-6.0, 9.6)
	*P* trend^b^	0.14	0.41	**0.036**
B: +BMI-adjusted	Shorter	2.3 (-0.2, 4.9)	2.9 (-0.3, 6.3)	-5.0 (-11.2, 1.3)
	About the same	0.0 (baseline)	0.0 (baseline)	0.0 (baseline)
	Taller	-0.3 (-2.5, 2.1)	1.0 (-2.0, 4.0)	5.6 (-0.5, 11.9)
	*P* trend^b^	0.065	0.30	**0.002**
C: +weight age 11 years	Shorter	1.5 (-1.0, 4.1)	1.9 (-1.3, 5.2)	-4.3 (-10.6, 2.2)
	About the same	0.0 (baseline)	0.0 (baseline)	0.0 (baseline)
	Taller	0.3 (-2.0, 2.7)	1.7 (-1.3, 4.8)	4.9 (-1.3, 11.2)
	*P* trend^b^	0.40	0.99	**0.010**

^a^Differences derived with respect to reference levels: 25% for percentage density, 30 cm^2^ for dense area and 110 cm^2^ for non-dense area. Models defined as A: analyses adjusted for age at mammogram (47–50, 50–54, 55–59 (baseline), 60–64, 65–69, 70–73 years), duration of oral contraception use (never (baseline), <5, 10–14, ≥15 years, not known), postmenopausal hormone treatment (never (baseline), former, current/<5, current/5–9, current/≥10 years duration), menopausal status and time since menopause (<5 (baseline), 10–14, 15–19, ≥20, unknown years postmenopausal, not postmenopausal), age at first birth and parity (nulliparous, 10–24 years/1–2, 10–24 years/≥3, 25–29 years/1–2 (baseline), 25–29 years/≥3, 30 years/≥1), alcohol units (none (baseline), 1–4 to ≥25, in 5-unit increments), physical activity (<31 (baseline), 32–55, 56–88, ≥88 metabolic energy equivalents/h/week); B: adjusted for covariates in model A plus body mass index (BMI) (<20.0 (baseline) to >35.0, in 2.5 kg/m^2^ increments); C: adjusted for covariates in model B plus weight compared with peers at age 11 years (thinner (baseline), about the same, heavier, unknown). ^b^ *P* trend for linear regression fitted through categories of exposure. Increase or decrease in category of height compared with peers between ages 7 and 11 years

The *p*-values in bold are those with *p*<0.05

In analyses of pubertal variables, age at thelarche was significantly positively associated with percentage density, but not with absolute dense area, in the basic model, but there was no association after taking into account adult adiposity (Table 4). However, there was an inverse association with non-dense area which remained statistically significant in models accounting for adiposity in adulthood and childhood. A later age at menarche and age at which regular cycles were established was associated with increased percentage and absolute dense area in models with and without adult BMI, which were no longer significant after taking into account childhood adiposity. There was no association with the age at which participants reported that they had reached their adult height (Additional file 1: Table S6).

**Table 4:** Difference in adult mammographic density parameters across categories of age at onset of pubertal stages

		Mammographic density parameters		
			Absolute area	
		Percent density difference,	Dense area	Non-dense area
Age at pubertal stage	Category	percentage points (95% CI)^a^	difference, cm^2^ (95% CI)^a^	difference, cm^2^ (95% CI)^a^
*Age at thelarche, years*				
A:	≤10	0.0 (baseline)	0.0 (baseline)	0.0 (baseline)
	11–12	2.6 (-1.2-6.7)	0.8 (-3.6, 5.5)	-8.8 (-19.3, 2.4)
	≥13	5.7 (1.6-10.1)	1.7 (-2.8, 6.6)	-23.1 (-33.1, -12.5)
	*P* trend^b^	**0.002**	0.42	**<0.001**
B: +BMI-adjusted	≤10	0.0 (baseline)	0.0 (baseline)	0.0 (baseline)
	11–12	1.0 (-2.4, 4.6)	0.0 (-4.2, 4.6)	-3.7 (-12.2, 5.1)
	≥13	2.2 (-1.4, 6.0)	-0.1 (-4.5, 4.6)	-11.7 (-20.1, -3.0)
	*P* trend^b^	0.20	0.95	**0.002**
C: +weight age 11 years	≤10	0.0 (baseline)	0.0 (baseline)	0.0 (baseline)
	11–12	0.2 (-3.1, 3.8)	-1.0 (-5.1, 3.5)	-3.0 (-11.5, 5.9)
	≥13	0.3 (-3.3, 4.1)	-2.5 (-6.8, 2.2)	-10.0 (-18.8, -0.7)
	*P* trend^b^	0.88	0.24	**0.012**
*Age at menarche, years*				
A:	≤12	0.0 (baseline)	0.0 (baseline)	0.0 (baseline)
	13–14	4.7 (2.1, 7.3)	3.5 (0.6, 6.6)	-12.7 (-19.5, -5.7)
	≥15	6.9 (2.7, 11.3)	5.7 (1.0, 10.8)	-17.7 (-28.1, -6.8)
	*P* trend^b^	**<0.001**	**0.003**	**<0.001**
B: +BMI-adjusted	≤12	0.0 (baseline)	0.0 (baseline)	0.0 (baseline)
	13–14	2.9 (0.6, 5.2)	2.9 (0.0, 5.9)	-5.2 (-10.8, 0.6)
	≥15	3.2 (-0.4, 7.1)	4.2 (-0.4, 9.1)	-3.1 (-12.1, 6.2)
	*P* trend^b^	**0.014**	**0.023**	0.18
C: +weight age 11 years	≤12	0.0 (baseline)	0.0 (baseline)	0.0 (baseline)
	13–14	2.0 (-0.3, 4.4)	1.7 (-1.1, 4.7)	-4.0 (-9.8, 1.9)
	≥15	1.8 (-1.8, 5.6)	2.3 (-2.2, 7.1)	-1.4 (-10.6, 8.2)
	*P* trend^b^	0.13	0.20	0.40
*Age at regular cycles, years*				
A:	≤12	0.0 (baseline)	0.0 (baseline)	0.0 (baseline)
	13–14	5.2 (1.9, 8.7)	5.4 (1.3, 9.7)	-10.0 (-18.8, -0.8)
	≥15	6.4 (2.5, 10.5)	5.3 (0.7, 10.3)	-12.4 (-22.4, -2.0)
	*P* trend^b^	**<0.001**	**0.013**	**0.014**
B: +BMI-adjusted	≤12	0.0 (baseline)	0.0 (baseline)	0.0 (baseline)
	13–14	3.3 (0.3, 6.5)	4.6 (0.6, 8.8)	-2.2 (-9.8, 5.6)
	≥15	4.3 (0.8, 8.1)	4.4 (-0.1, 9.2)	-4.3 (-12.8, 4.5)
	*P* trend^b^	**0.010**	**0.036**	0.33
C: +weight age 11 years	≤12	0.0 (baseline)	0.0 (baseline)	0.0 (baseline)
	13–14	2.5 (-0.6, 5.7)	3.6 (-0.4, 7.9)	-0.5 (-8.2, 7.5)
	≥15	3.4 (-0.1, 7.1)	3.4 (-1.1, 8.2)	-2.6 (-11.2, 6.5)
	*P* trend^b^	0.051	0.12	0.59

^a^Differences derived with respect to reference levels: 25% for percentage density, 30 cm^2^ for dense area and 110 cm^2^ for non-dense area. Models defined as A: analyses adjusted for age at mammogram (47–50, 50–54, 55–59 (baseline), 60–64, 65–69, 70–73 years), duration of oral contraception use (never (baseline), <5, 10–14, ≥15 years, not known), postmenopausal hormone treatment (never (baseline), former, current/<5, current/5–9, current/≥10 years duration), menopausal status and time since menopause (<5 (baseline), 10–14, 15–19, ≥20, unknown years postmenopausal, not postmenopausal), age at first birth and parity (nulliparous, 10–24 years/1–2, 10–24 years/≥3, 25–29 years/1–2 (baseline), 25–29 years/≥3, 30 years/≥1), alcohol units (none (baseline), 1–4 to ≥25, in 5-unit increments), physical activity (<31 (baseline), 32–55, 56–88, ≥88 metabolic energy equivalents/h/week); B: adjusted for covariates in model A plus body mass index (BMI) (<20.0 (baseline) to >35.0, in 2.5 kg/m^2^ increments); C: adjusted for covariates in model B plus weight compared with peers at age 11 years (thinner (baseline), about the same, heavier, not known). ^b^ *P* trend for linear regression fitted through categories of exposure

The *p*-values in bold are those with *p*<0.05

There was evidence of association between a longer time interval between thelarche and menarche and increased mammographic dense area and between a longer time interval from thelarche to establishment of regular periods and increased mammographic dense area, even after adjusting for childhood adiposity (Table 5). There was a similar tendency for association with percentage density but this was not statistically significant. The association with the interval from thelarche to menarche remained statistically significant after controlling for age at thelarche (*p* trend = 0.020), menarche (*p* trend = 0.037), or total breast area (*p* trend = 0.023) in a model accounting for adult and childhood adiposity. Likewise, the association with the time from thelarche to establishment of regular periods remained significant after controlling for age at thelarche (*p* trend = 0.035) or became borderline significant after controlling for age at the establishment of regular cycles (*p* trend = 0.060) or for total breast area (*p* trend = 0.048) (not shown). Density was not associated with the interval between age at menarche and establishment of regular cycles (Additional file 1: Table S7), or the interval between thelarche and the age at which the participant reached adult height or between menarche and age of attainment of adult height after accounting for BMI (Additional file 1: Table S8).

**Table 5:** Difference in adult mammographic density parameters across categories of timing between pubertal stages

		Mammographic density parameters		
			Absolute area	
		Percent density difference,	Dense area	Non-dense area
Interval between pubertal stages	Category	percentage points (95% CI)^a^	difference, cm^2^ (95% CI)^a^	difference, cm^2^ (95% CI)^a^
*Thelarche to menarche, years*				
A:	<0	-0.4 (-4.8, 4.5)	-0.3 (-5.6, 5.5)	-3.9 (-17.6, 10.6)
	0	0.0 (baseline)	0.0 (baseline)	0.0 (baseline)
	1	2.2 (-0.7, 5.2)	4.1 (0.5, 7.8)	-0.1 (-8.7, 8.8)
	≥2	2.0 (-1.9, 6.2)	4.0 (-0.7, 9.1)	-1.3 (-12.6, 10.8)
	*P* trend^b^	0.14	**0.022**	0.87
B: +BMI-adjusted	<0	-0.5 (-4.5, 3.9)	-0.7 (-5.8, 5.0)	-4.7 (-15.3, 6.4)
	0	0.0 (baseline)	0.0 (baseline)	0.0 (baseline)
	1	1.7 (-0.9,4.5)	4.1 (0.6, 7.8)	1.9 (-4.8, 8.8)
	≥2	2.1 (-1.5, 5.8)	3.8 (-0.8, 8.7)	-1.7 (-10.5, 7.5)
	*P* trend^b^	0.13	**0.019**	0.64
C: +weight age 11 years	<0	-1.3 (-5.3, 3.0)	-1.7 (-6.8, 3.8)	-3.5 (-14.2, 7.7)
	0	0.0 (baseline)	0.0 (baseline)	0.0 (baseline)
	1	1.7 (-1.0, 4.4)	4.0 (0.6, 7.7)	2.0 (-4.8, 8.9)
	≥2	1.8 (-1.7, 5.5)	3.4 (-1.1, 8.3)	-1.3 (-10.2, 7.9)
	*P* trend^b^	0.10	**0.013**	0.69
*Thelarche to regular cycles, years*				
A:	<0	-5.6 (-11.4, 1.1)	-7.8 (-14.7, 0.3)	6.2 (-14.4, 28.9)
	0	0.0 (baseline)	0.0 (baseline)	0.0 (baseline)
	1	1.7 (-2.2, 5.9)	1.8 (-2.9, 6.9)	-2.5 (-13.6, 9.3)
	≥2	1.6 (-2.3, 5.9)	2.6 (-2.3, 7.8)	1.6 (-10.0, 13.8)
	*P* trend^b^	0.083	**0.039**	0.97
B: +BMI-adjusted	<0	-2.9 (-8.6, 3.7)	-6.2 (-13.2, 2.0)	-7.3 (-23.0, 9.7)
	0	0.0 (baseline)	0.0 (baseline)	0.0 (baseline)
	1	1.8 (-1.8, 5.6)	1.8 (-2.9, 6.7)	-3.5 (-12.3, 5.8)
	≥2	2.5 (-1.2, 6.4)	3.3 (-1.5, 8.5)	-1.5 (-10.6, 8.1)
	*P* trend^b^	0.068	**0.029**	0.87
C: +weight age 11 years	<0	-3.6 (-9.2, 2.9)	-7.2 (-14.1, 0.8)	-6.6 (-22.5, 10.5)
	0	0.0 (baseline)	0.0 (baseline)	0.0 (baseline)
	1	1.8 (-1.8, 5.6)	1.8 (-2.8, 6.7)	-3.4 (-12.3, 5.9)
	≥2	2.3 (-1.4, 6.2)	3.0 (-1.8, 8.1)	-1.4 (-10.5, 8.2)
	*P* trend^b^	0.064	**0.028**	0.89

^a^Differences derived with respect to reference levels: 25% for percentage density, 30 cm^2^ for dense area and 110 cm^2^ for non-dense area. Models defined as A: analyses adjusted for age at mammogram (47–50, 50–54, 55–59 (baseline), 60–64, 65–69, 70–73 years), duration of oral contraception use (never (baseline), <5, 10–14, ≥15 years, not known), postmenopausal hormone treatment (never (baseline), former, current/<5, current/5–9, current/≥10 years duration), menopausal status and time since menopause (<5 (baseline), 10–14, 15–19, ≥20, unknown years postmenopausal, not postmenopausal), age at first birth and parity (nulliparous, 10–24 years/1–2, 10–24 years/≥3, 25–29 years/1–2 (baseline), 25–29 years/≥3, 30 years/≥1), alcohol units (none (baseline), 1–4 to ≥25, in 5-unit increments), physical activity (<31 (baseline), 32–55, 56–88, ≥88 metabolic energy equivalents/h/week); B: adjusted for covariates in model A plus body mass index (BMI) (<20.0 (baseline) to >35.0, in 2.5 kg/m^2^ increments); C: adjusted for covariates in model B plus weight compared with peers at age 11 years (thinner (baseline), about the same, heavier, not known). ^b^ *P* trend for linear regression fitted through categories of exposure

The *p*-values in bold are those with *p*<0.05

## Discussion

This is, to our knowledge, the first study to investigate pubertal stages other than age at menarche with respect to quantitatively assessed adult breast density. We found evidence of association between later onset of pubertal stages, in particular age at menarche and age at establishment of regular cycles, and increased mammographic density. This study also showed that girls who were heavier than their peers in childhood had significantly lower mammographic density in adulthood, even after adjusting for adult adiposity, which correlates with childhood adiposity. As expected, increased childhood weight predicted earlier pubertal onset, and we found that the positive association of delayed puberty with density appeared to be driven by childhood weight. However, we observed a tendency for increased mammographic dense area in women reporting longer intervals between thelarche and menarche, and between thelarche and regular cycles, which was independent of the effect of age at onset and it is of interest that a prolonged pubertal tempo has also been implicated in breast cancer risk in a previous publication from our study [7].

An inverse association of childhood weight with adult mammographic density is supported by most, but not all, previous studies [19]. A review suggests that evidence of such an association is stronger in postmenopausal than in premenopausal women [19]; our study included too few premenopausal women to analyse the data by menopausal status. While these studies investigated adult density later in life, the inverse association between body size and density has also been demonstrated with a measure of density at younger ages, using magnetic resonance imaging [24, 25]. The biological mechanism through which increased adiposity is associated with mammographic density is possibly through lower insulin-like growth factor (IGF)-I in heavier girls [26, 27], or a protective function of adipocytes [19]. There is increasing evidence that heavier body weight in childhood and adolescence is also inversely associated with subsequent breast cancer risk [28] and it seems likely that this may in part be through an effect of adiposity on breast density.

Our study suggested an inverse association between percentage density and height at age 7 years and no association between percentage density and height at age 11 years, contradicting the two previous studies of similar design, in which there was higher percentage density in those who reported to have been taller than their peers in childhood [13, 18]. Our findings are more compatible with those of a large study showing an inverse association between having mixed/dense breasts in adulthood and measured height at prepubertal and peripubertal ages [29]. Height at both ages was positively associated with non-dense area in our study, even after adjusting for adult adiposity. This finding could reflect that taller girls had larger overall breast size (non-dense area being the largest component), or possibly residual confounding by BMI, as non-dense area and BMI are strongly correlated. In contrast to our lack of association with age at reaching adult height or having had a relative growth spurt, a previous study reported an increase in Wolfe-grade density with greater velocity of growth in height at ages 11–15 years and 15 years to adulthood, based on measured height [14]. Studies have not consistently shown an association between adult height and breast density, with some reporting positive [18, 30], some weak and some no association [31–33] with percentage density. Whether childhood or adult height is a determinant of breast density is therefore still not entirely clear.

Breast density has been hypothesised to represent the cumulative exposure of tissue to hormones and growth factors that stimulate cell division and it has been proposed that tissue composition reflects such exposures at young ages during the greatest susceptibility of the breast according to the Pike model [8, 34]. The development of the human breast is a process that is initiated in utero, but the main growth spurt occurs with the formation of lobules during puberty (i.e. at thelarche). Increased estradiol production is thought to be largely responsible for breast development in pubescent girls, and increases in oestradiol levels have been demonstrated around the onset of breast development [35]. The pubertal stage of peak growth, when linear height increase is accelerated, is accompanied by high levels of growth hormones, sex hormones and IGF-I [36, 37]. Around menarche the rate at which breast ducts grow and proliferate increases [38]. An earlier age at which regular menses are established is thought to be associated with higher cumulative exposure to ovarian hormones, as women with irregular cycles spend relatively less time in the luteal phase of the menstrual cycle when hormone levels are highest [39].

Body adiposity is a strong predictor of pubertal onset, possibly mediated by leptin. Age at thelarche normally indicates gonadotropin-driven ovarian oestrogen production, but it has been postulated that breast development in obese girls is a consequence of aromatisation from adrenal androgen precursors to oestrogens in adipose tissue, which might explain the fact that early onset of breast development appears to be compensated by slower progression to menarche [40]. Increased levels of total and free testosterone, lower levels of sex hormone binding globulin (SHBG) and higher levels of fasting insulin have been reported in peripubertally obese girls [41] and lower oestradiol levels in heavier girls compared with lighter girls around the time of thelarche [35]. Few studies have investigated the role of peripubertal hormone levels on determination of adult mammographic density. One study showed that higher pre-menarcheal SHBG or dehydroepiandrosterone sulfate (DHEAS), but not oestradiol, was associated with increased mammographic dense area [42], whereas in another study tall girls treated with high-dose oestrogen to accelerate puberty were reported to have lower mammographic dense area in adulthood [43].

After controlling for adult adiposity, we found that women with later onset of first or regular menses had higher mammographic density than those with early onset. Our finding is broadly in line with previous studies that after controlling for adiposity, have shown significant positive association [10, 14–16] between density and menarche, although some studies have reported no association [9, 11–13]. Positive associations with pubertal onset appear to be largely a consequence of increased childhood body weight being a strong predictor of earlier pubertal onset, however, because we did not observe significant associations independent of relative childhood weight. We found that an early age at thelarche was associated with lower adult density and that this finding was in part explained by adult adiposity. This is supported by a study reporting less dense breasts measured qualitatively by the Wolfe grade in girls with signs of breast development at age 11 years [14], and another study in young girls, with breast density measured by dual-energy absorptiometry, which showed that the major determinants of breast density during puberty are body fat, achievement of menarche and Tanner breast stage [44].

Our analyses suggest that previously reported associations between breast cancer risk and earlier thelarche, menarche, regular periods or age that adult height is reached [7], are unlikely to be mediated by mammographic density. In fact, the associations we observed were in the opposite direction to that related to breast cancer (i.e. later pubertal onset was positively associated with density but is thought to be inversely associated with breast cancer risk). These findings imply that in analyses of the effect of age at menarche on breast cancer risk, controlling for density would strengthen the association. A prolonged interval between breast development and onset of menarche or regular periods appeared to increase dense breast area in our study, which could possibly be due to prolonged exposure of breast tissue to hormones and growth factors, but could also be due to chance or residual confounding, and would therefore need to be investigated in further studies.

Our study has the strength that subjects were selected from a prospective study with comprehensive information on breast cancer risk factors. A limitation is that the pubertal and weight variables that we collected were self-reported. Also, BMI was assessed at baseline and was not available at the exact time of mammography, and we were unable to collect exact weights in childhood and our proxy variables of weight in childhood relative to peers and the variable for growth spurt are therefore relatively crude measures. The accuracy of reporting of age at menarche and body size in childhood is thought to be reasonably good [45], but recall of the timing of the onset of breast growth, regular menses and age at attained adult height is likely to be less accurate. It is unlikely that quality of recall is related to mammographic density measurement, however, and these variables previously showed significant associations with breast cancer risk in our prospective study, suggesting they are sufficiently discriminatory. We did not have information on peak growth but analysed age at attained adult height, with which it is correlated, as a proxy [46, 47].

## Conclusions

Adult mammographic density was inversely associated with weight compared to peers at ages 7 and 11 years, and was not independently associated with age at onset of pubertal stages. The role of a prolonged duration between breast development and onset of first or regular menses on breast density needs investigation in future studies.

## Additional file

Additional file 1: Supplementary tables. **Table S1.** Number of subjects included in analyses of categories of body size and pubertal factors. **Table S2.** Adjusted means of pubertal variables, anthropometric and mammographic density characteristics by weight compared with peers at age 11 years. **Table S3.** Adjusted means of pubertal variables, anthropometric and mammographic density characteristics by height compared with peers at age 11 years. **Table S4.** Correlations between pubertal factors and adult body mass index. **Table S5.** Difference in adult mammographic density parameters in relation to change in height compared with peers between ages 7 and 11 years. **Table S6.** Difference in adult mammographic density parameters across categories of age at reaching adult height. **Table S7.** Difference in adult mammographic density parameters across categories of time interval between menarche and regular cycles. **Table S8.** Difference in adult mammographic density parameters in relation to time interval between thelarche or menarche and age at reaching adult height. (PDF 417 kb)

## Abbreviations

BMI: Body mass index
IGF: Insulin-like growth factor
SHBG: Sex hormone-binding globulin
UK: United Kingdom
