# Supplementary material for: Childhood body size and pubertal timing in relation to adult mammographic density phenotype
Source: Breast Cancer Res. 2017 Feb 7;19:13. doi: 10.1186/s13058-017-0804-y (PMC5297131; doi:10.1186/s13058-017-0804-y)
Supplement: Additional file 1: — Supplementary tables. Table S1. Number of subjects included in analyses of categories of body size and pubertal factors. Table S2. Adjusted means of pubertal variables, anthropometric and mammographic density characteristics by weight compared with peers at age 11 years. Table S3. Adjusted means of pubertal variables, anthropometric and mammographic density characteristics by height compared with peers at age 11 years. Table S4. Correlations between pubertal factors and adult body mass index. Table S5. Difference in adult mammographic density parameters in relation to change in height compared with peers between ages 7 and 11 years. Table S6. Difference in adult mammographic density parameters across categories of age at reaching adult height. Table S7. Difference in adult mammographic density parameters across categories of time interval between menarche and regular cycles. Table S8. Difference in adult mammographic density parameters in relation to time interval between thelarche or menarche and age at reaching adult height. (PDF 417 kb) [file 13058_2017_804_MOESM1_ESM.pdf]

Childhood body size and pubertal timing in relation to adult mammographic density phenotype

Supplementary tables

Table S1: Number of subjects included in analyses of categories of body size and pubertal factors.

Factor	Categories, numbers (%)				Missing or not applicable
	Thinner	*About the same*	*Heavier*		
Weight at age 7 years	311 (28.8)	633 (58.7)	135 (12.5)		26
Weight at age 11 years	299 (27.4)	589 (53.9)	204 (18.7)		13
	Shorter	*About the same*	*Taller*		
Height at age 7 years	245 (23.0)	542 (50.8)	280 (26.2)		38
Height at age 11 years	281 (26.0)	458 (42.3)	343 (31.7)		23
	≤10 years	*11-12 years*	*≥13 years*		
Age at thelarche	111 (12.6)	415 (47.0)	357 (40.4)		222
	≤12 years	*13-14 years*	*≥15 years*		
Age at menarche	454 (45.6)	435 (43.7)	107 (10.7)		109
Age at regular cycles	243 (36.3)	265 (40.0)	162 (24.2)		435
	≤14 years	*15-16 years*	*≥17 years*		
Age reached adult height	192 (32.1)	255 (42.6)	152 (25.4)		506
	<0 years	*0 years*	*1 years*	*≥2 years*	
Thelarche to menarche	73 (9.0)	315 (38.7)	310 (38.1)	116 (14.3)	291
Thelarche to regular cycles	31 (5.3)	141 (24.1)	223 (38.1)	190 (32.5)	520
Menarche to regular cycles		395 (59.0)	196 (29.3)	79 (11.8)	435
	<2 years	*2-3 years*	*≥4 years*		
Thelarche to adult height	96 (17.6)	209 (38.4)	239 (43.9)		561
Menarche to adult height	169 (30.9)	195 (35.6)	184 (33.6)		557

Table S2: Adjusted means of pubertal variables, anthropometric and mammographic density characteristics by weight compared with peers at age 11 years

	Weight at age 11 years relative to peers			
	Thinner	About the same	Heavier	
	Mean	Mean	Mean	P trend (c)
Pubertal variables (a)				
Age at thelarche, years	12.9	12.0	11.4	<0.001
Age at menarche, years	13.3	12.6	12.1	<0.001
Age at regular cycles, years	14.3	13.7	13.4	0.004
Age reached attained height, years	15.9	15.3	15.1	0.001
Thelarche to menarche, years	0.51	0.62	0.68	0.12
Thelarche to regular cycles, years	1.22	1.28	1.58	0.15
Thelarche to attained height, years	3.07	3.40	3.97	0.001
Menarche to regular cycles, years	0.66	0.65	0.86	0.29
Menarche to attained height, years	2.58	2.72	3.15	0.037
Adult anthropometrics (a)				
Attained height, cms	163.6	162.6	163.8	0.97
Body mass index at mammogram, kg/m^2	24.4	25.8	27.9	<0.001
Mammographic density, mean (b)				
Percentage density, %	23.8	21.3	17.3	<0.001
Absolute dense area, cm^2	29.3	25.9	20.7	<0.001
Absolute nondense area, cm^2	94.5	97.5	103.5	0.017
Absolute total breast area, cm^2	129.4	129.1	130.3	0.83

(a) Adjusted mean for age at mammogram of 58 years

(b) Adjusted mean (back-transformed to ordinary scale) for age at mammogram of 58 years and BMI at study entry of 25 kg/m^2

(c) From linear regression model per category of weight compared with peers at age 11 years

Table S3: Adjusted means of pubertal variables, anthropometric and mammographic density characteristics by height compared with peers at age 11 years

	Height at age 11 years relative to peers			
	Shorter	About the same	Taller	
	Mean	Mean	Mean	P trend (c)
Pubertal variables (a)				
Age at thelarche, years	12.6	12.1	11.8	<0.001
Age at menarche, years	13.1	12.7	12.5	<0.001
Age at regular cycles, years	14.2	13.8	13.5	0.019
Age reached attained height, years	15.7	15.6	15.1	0.003
Thelarche to menarche, years	0.59	0.59	0.62	0.79
Thelarche to regular cycles, years	1.38	1.41	1.13	0.20
Thelarche to attained height, years	3.18	3.63	3.39	0.49
Menarche to regular cycles, years	0.72	0.75	0.60	0.37
Menarche to attained height, years	2.56	3.01	2.69	0.73
Adult anthropometrics (a)				
Attained height, cms	157.7	162.7	168.1	<0.001
Body mass index at mammogram, kg/m^2	25.7	26.1	25.5	0.46
Mammographic density, mean (b)				
Percentage density, %	22.5	21.0	20.7	0.16
Absolute dense area, cm^2	27.0	25.1	26.4	0.72
Absolute nondense area, cm^2	93.2	96.7	102.6	0.003
Absolute total breast area, cm^2	125.8	127.6	135.2	0.002

(a) Adjusted mean for age at mammogram of 58 years

(b) Adjusted mean (back-transformed to ordinary scale) for age at mammogram of 58 years and BMI at study entry of 25 kg/m^2

(c) From linear regression model per category of height compared with peers at age 11 years

Table S4: Correlations between pubertal factors and adult body mass index

	Age at thelarche	Age at menarche	Age at regular cycles	Age reached adult height	Attained height	Adult BMI
Age at thelarche	1.00					
Age at menarche	0.74	1.00				
Age at regular periods	0.34	0.51	1.00			
Age reached attained height	0.27	0.30	0.17	1.00		
Attained height	0.09	0.11	0.05	0.22	1.00	
Adult BMI	-0.16	-0.15	-0.08	0.003	-0.18	1.00

Table S5: Difference in adult mammographic density parameters in relation to change in height compared with peers between age 7 and 11 years.

		Mammographic density parameters		
			Absolute area	
		Percent density	Dense area	Non-dense area
Change in relative height age 7 to 11 years (c)	Category	Difference, percentage points (95% CI)(a)	Difference, cm^2 (95% CI)(a)	Difference, cm^2 (95% CI)(a)
A:	Decrease	-4.8 (-8.9, -0.4)	-3.4 (-8.4, 2.1)	18.8 (3.7, 34.9)
	About the same	0.0 (baseline)	0.0 (baseline)	0.0 (baseline)
	Increase	-1.3 (-5.0, 2.7)	-1.2 (-5.5, 3.6)	8.9 (-3.3, 21.7)
	P trend (b)	0.36	0.66	0.59
B: +BMI adjusted	Decrease	-3.6 (-7.4, 0.6)	-2.5 (-7.5, 3.0)	13.0 (1.3, 25.3)
	About the same	0.0 (baseline)	0.0 (baseline)	0.0 (baseline)
	Increase	-0.7 (-4.1, 3.0)	-0.9 (-5.2, 3.8)	6.3 (-3.1, 16.2)
	P trend (b)	0.41	0.75	0.65
C: +weight age 11 years	Decrease	-3.6 (-7.4, 0.5)	-2.5 (-7.5, 2.9)	13.2 (1.6, 25.4)
	About the same	0.0 (baseline)	0.0 (baseline)	0.0 (baseline)
	Increase	-0.3 (-3.7, 3.4)	-0.3 (-4.6, 4.3)	6.2 (-3.3, 16.0)
	P trend (b)	0.30	0.61	0.62

(a) Differences derived with respect to reference levels: 25% for percentage density, 30 cm^2 for dense area and 110 cm^2 for nondense area. Models defined as follows: Model A: Analyses adjusted for age at mammogram (47-50, 50-54, 55-59 (baseline), 60-64, 65-69, 70-73 years), duration of oral contraception use (never (baseline), <5, 10-14, ≥15 years, not known), postmenopausal hormone treatment (never (baseline), former, current/<5, current/5-9, current/≥10 years duration), menopausal status and time since menopause (<5 (baseline), 10-14, 15-19, ≥20, unknown years postmenopausal, not postmenopausal), age at first birth and parity (nulliparous, 10-24y/1-2, 10-24y/≥3, 25-29y/1-2 (baseline), 25-29y/≥3, 30y/≥1), alcohol units (none (baseline), 1-4 to ≥25, in 5-unit increments), physical activity (<31 (baseline), 32-55, 56-88, ≥88 MET-hr/wk)
Model B: adjusted for covariates in model A plus BMI (<20.0 (baseline) to >35.0, in 2.5 kg/m^2 increments)
Model C: adjusted for covariates in model B plus weight compared with peers at age 11 years (thinner (baseline), about the same, heavier, not known)

(b) P trend for linear regression fitted through categories of exposure

(c) Increase or decrease in category of height compared with peers between ages 7 and 11 years

Table S6: Difference in adult mammographic density parameters across categories of age at reaching adult height

Age at pubertal stage	Category	Mammographic density parameters: Percent density — Difference, percentage points (95% CI)(a)	Absolute area: Dense area — Difference, cm^2 (95% CI)(a)	Absolute area: Non-dense area — Difference, cm^2 (95% CI)(a)
Age reached adult height, years				
A:	≤14	0.0 (baseline)	0.0 (baseline)	0.0 (baseline)
	15-16	-0.5 (-3.9, 3.0)	-0.6 (-4.5, 3.5)	4.3 (-6.3, 15.4)
	≥17	0.9 (-2.9, 5.0)	1.3 (-3.2, 6.0)	-0.4 (-11.9, 11.8)
	P trend (b)	0.70	0.62	0.99
B: +BMI adjusted	≤14	0.0 (baseline)	0.0 (baseline)	0.0 (baseline)
	15-16	-1.2 (-4.2, 1.9)	-1.1 (-4.8, 2.9)	7.4 (-1.0, 16.2)
	≥17	0.6 (-2.9, 4.3)	0.5 (-3.7, 5.2)	0.0 (-9.1, 9.5)
	P trend (b)	0.80	0.85	0.89
C: +weight age 11 years	≤14	0.0 (baseline)	0.0 (baseline)	0.0 (baseline)
	15-16	-1.7 (-4.7, 1.5)	-1.5 (-5.2, 2.5)	8.6 (0.1, 17.5)
	≥17	0.0 (-3.4, 3.7)	0.0 (-4.2, 4.6)	1.3 (-7.9, 10.9)
	P trend (b)	0.94	0.97	0.69

(a) Differences derived with respect to reference levels: 25% for percentage density, 30 cm^2 for dense area and 110 cm^2 for nondense area. Models defined as follows: Model A: Analyses adjusted for age at mammogram (47-50, 50-54, 55-59 (baseline), 60-64, 65-69, 70-73 years), duration of oral contraception use (never (baseline), <5, 10-14, ≥15 years, not known), postmenopausal hormone treatment (never (baseline), former, current/<5, current/5-9, current/≥10 years duration), menopausal status and time since menopause (<5 (baseline), 10-14, 15-19, ≥20, unknown years postmenopausal, not postmenopausal), age at first birth and parity (nulliparous, 10-24y/1-2, 10-24y/≥3, 25-29y/1-2 (baseline), 25-29y/≥3, 30y/≥1), alcohol units (none (baseline), 1-4 to ≥25, in 5-unit increments), physical activity (<31 (baseline), 32-55, 56-88, ≥88 MET-hr/wk)
Model B: adjusted for covariates in model A plus BMI (<20.0 (baseline) to >35.0, in 2.5 kg/m^2 increments)
Model C: adjusted for covariates in model B plus weight compared with peers at age 11 years (thinner (baseline), about the same, heavier, not known)

(b) P trend for linear regression fitted through categories of exposure

Table S7: Difference in adult mammographic density parameters across categories of time interval between menarche and regular cycles

		Mammographic density parameters		
			Absolute area	
		Percent density	Dense area	Non-dense area
Time interval	Category	Difference, percentage points (95% CI)(a)	Difference, cm^2 (95% CI)(a)	Difference, cm^2 (95% CI)(a)
Menarche to regular cycles, years				
A:	0	0.0 (baseline)	0.0 (baseline)	0.0 (baseline)
	1	-1.1 (-4.0, 2.1)	-2.5 (-6.0, 1.4)	0.27 (-9.0, 9.9)
	≥2	-0.5 (-4.8, 4.1)	-0.1 (-5.3, 5.6)	6.5 (-6.9, 20.8)
	P trend (b)	0.63	0.55	0.44
B: +BMI adjusted	0	0.0 (baseline)	0.0 (baseline)	0.0 (baseline)
	1	-2.0 (-4.7, 0.9)	-2.8 (-6.3, 0.9)	5.1 (-2.6, 13.0)
	≥2	-0.4 (-4.3, 3.9)	0.2 (-5.0, 5.8)	5.9 (-5.1, 17.3)
	P trend (b)	0.46	0.55	0.17
C: +weight age 11 years	0	0.0 (baseline)	0.0 (baseline)	0.0 (baseline)
	1	-1.9 (-4.7, 0.9)	-2.7 (-6.2, 1.0)	5.2 (-2.5, 13.1)
	≥2	-0.4 (-4.3, 3.8)	0.0 (-5.1, 5.6)	5.8 (-5.1, 17.1)
	P trend (b)	0.46	0.54	0.16

(a) Differences derived with respect to reference levels: 25% for percentage density, 30 cm^2 for dense area and 110 cm^2 for nondense area. Models defined as follows: Model A: Analyses adjusted for age at mammogram (47-50, 50-54, 55-59 (baseline), 60-64, 65-69, 70-73 years), duration of oral contraception use (never (baseline), <5, 10-14, ≥15 years, not known), postmenopausal hormone treatment (never (baseline), former, current/<5, current/5-9, current/≥10 years duration), menopausal status and time since menopause (<5 (baseline), 10-14, 15-19, ≥20, unknown years postmenopausal, not postmenopausal), age at first birth and parity (nulliparous, 10-24y/1-2, 10-24y/≥3, 25-29y/1-2 (baseline), 25-29y/≥3, 30y/≥1), alcohol units (none (baseline), 1-4 to ≥25, in 5-unit increments), physical activity (<31 (baseline), 32-55, 56-88, ≥88 MET-hr/wk) Model B: adjusted for covariates in model A plus BMI (<20.0 (baseline) to >35.0, in 2.5 kg/m^2 increments) Model C: adjusted for covariates in model B plus weight compared with peers at age 11 years (thinner (baseline), about the same, heavier, not known)

(b) P trend for linear regression fitted through categories of exposure

Table S8: Difference in adult mammographic density parameters in relation to time interval between thelarche or menarche and age at reaching adult height

		Mammographic density parameters		
			Absolute area	
		Percent density	Dense area	Non-dense area
Time interval	Category	Difference, percentage points (95% CI)(a)	Difference, cm^2 (95% CI)(a)	Difference, cm^2 (95% CI)(a)
Thelarche to reaching adult height, years				
A:	<2	0.0 (baseline)	0.0 (baseline)	0.0 (baseline)
	2-3	2.0 (-2.5, 6.9)	-0.3 (-5.2, 5.2)	-11.8 (-24.2, 1.4)
	≥4	-0.4 (-4.6, 4.1)	0.0 (-4.8, 5.4)	3.6 (-9.5, 17.3)
	P trend (b)	0.57	0.95	0.18
B: +BMI adjusted	<2	0.0 (baseline)	0.0 (baseline)	0.0 (baseline)
	2-3	-0.1 (-4.1, 4.2)	-1.3 (-6.1, 3.9)	-4.0 (-14.3, 6.9)
	≥4	-0.1 (-4.0, 4.0)	0.1 (-4.7, 5.2)	2.4 (-7.9, 13.2)
	P trend (b)	0.95	0.81	0.40
C: +weight age 11	<2	0.0 (baseline)	0.0 (baseline)	0.0 (baseline)
	2-3	0.2 (-3.8, 4.5)	-0.9 (-5.8, 4.3)	-4.2 (-14.5, 6.7)
	≥4	0.5 (-3.4, 4.7)	0.8 (-4.1, 6.1)	1.4 (-8.9, 12.2)
	P trend (b)	0.80	0.60	0.54
Menarche to reaching adult height, years				
A:	<2	0.0 (baseline)	0.0 (baseline)	0.0 (baseline)
	2-3	-4.0 (-7.4, -0.4)	-5.1 (-9.1, -0.9)	5.6 (-6.4, 18.2)
	≥4	-4.4 (-7.7, -0.8)	-3.8 (-7.8, 0.5)	13.9 (1.7, 26.7)
	P trend (b)	0.020	0.10	0.025
B: +BMI adjusted	<2	0.0 (baseline)	0.0 (baseline)	0.0 (baseline)
	2-3	-4.5 (-7.6, -1.2)	-5.6 (-9.4, -1.4)	7.0 (-2.6, 16.9)
	≥4	-3.3 (-6.5, 0.0)	-3.4 (-7.3, 0.9)	8.0 (-1.4, 17.7)
	P trend (b)	0.061	0.14	0.10
C: +weight age 11	<2	0.0 (baseline)	0.0 (baseline)	0.0 (baseline)
	2-3	-4.5 (-7.6, -1.2)	-5.5 (-9.3, -1.3)	7.3 (-2.3, 17.3)
	≥4	-3.1 (-6.3, 0.3)	-3.0 (-7.0, 1.3)	7.8 (-1.6, 17.6)
	P trend (b)	0.082	0.19	0.11

(a) Differences derived with respect to reference levels: 25% for percentage density, 30 cm^2 for dense area and 110 cm^2 for nondense area. Models defined as follows: Model A: Analyses adjusted for age at mammogram (47-50, 50-54, 55-59 (baseline), 60-64, 65-69, 70-73 years), duration of oral contraception use (never (baseline), <5, 10-14, ≥15 years, not known), postmenopausal hormone treatment (never (baseline), former, current/<5, current/5-9, current/≥10 years duration), menopausal status and time since menopause (<5 (baseline), 10-14, 15-19, ≥20, unknown years postmenopausal, not postmenopausal), age at first birth and parity (nulliparous, 10-24y/1-2, 10-24y/≥3, 25-29y/1-2 (baseline), 25-29y/≥3, 30y/≥1), alcohol units (none (baseline), 1-4 to ≥25, in 5-unit increments), physical activity (<31 (baseline), 32-55, 56-88, ≥88 MET-hr/wk) Model B: adjusted for covariates in model A plus BMI (<20.0 (baseline) to >35.0, in 2.5 kg/m^2 increments) Model C: adjusted for covariates in model B plus weight compared with peers at age 11 years (thinner (baseline), about the same, heavier, not known)

(b) P trend for linear regression fitted through categories of exposure
